# Old Lineages in a New Ecosystem: Diversification of Arcellinid Amoebae (Amoebozoa) and Peatland Mosses

**DOI:** 10.1371/journal.pone.0095238

**Published:** 2014-04-24

**Authors:** Omar Fiz-Palacios, Brian S. Leander, Thierry J. Heger

**Affiliations:** 1 Systematic Biology Program, Department of Organismal Biology, Evolutionary Biology Centre, Uppsala, Sweden; 2 Biodiversity Research Center, Departments of Zoology and Botany, University of British Columbia, Vancouver, BC, Canada; MRC Laboratory of Molecular Biology, United Kingdom

## Abstract

Arcellinid testate amoebae (Amoebozoa) form a group of free-living microbial eukaryotes with one of the oldest fossil records known, yet several aspects of their evolutionary history remain poorly understood. Arcellinids occur in a range of terrestrial, freshwater and even brackish habitats; however, many arcellinid morphospecies such as *Hyalosphenia papilio* are particularly abundant in *Sphagnum*-dominated peatlands, a relatively new ecosystem that appeared during the diversification of *Sphagnum* species in the Miocene (5–20 Myr ago). Here, we reconstruct divergence times in arcellinid testate amoebae after selecting several fossils for clock calibrations and then infer whether or not arcellinids followed a pattern of diversification that parallels the pattern described for *Sphagnum*. We found that the diversification of core arcellinids occurred during the Phanerozoic, which is congruent with most arcellinid fossils but not with the oldest known amoebozoan fossil (i.e. at ca. 662 or ca. 750 Myr). Overall, *Sphagnum* and the Hyalospheniidae exhibit different patterns of diversification. However, an extensive molecular phylogenetic analysis of distinct clades within *H. papilio* species complex demonstrated a correlation between the recent diversification of *H. papilio*, the recent diversification of *Sphagnum* mosses, and the establishment of peatlands.

## Introduction

The Amoebozoa is a sister clade to animals and fungi and is considered to reflect one of the most ancient lineages of eukaryotes [1], [2]. Amoebozoans are unicellular eukaryotes, but a few taxa such as the model species *Dictyostelium discoideum* can form multicellular structures when nutrient conditions are limiting. Although the phylogenetic relationships within the Amoebozoa are far from being fully resolved, one subgroup, the Tubulinea, has been consistently recovered with strong statistical support [3]–[5], and includes one of the most species-rich groups of amoebozoans, namely the Arcellinida (>1000 species) [3], [4], [6]. Arcellinid amoebae occur in a range of terrestrial, freshwater and even brackish habitats [7], [8], and are particularly diverse and abundant in *Sphagnum*-dominated peatlands [9]–[11]. Several testate amoebae within the Hyalospheniidae, such as *Nebela carinata* and *Hyalosphenia papilio*, are very abundant in *Sphagnum* mosses, but they can be also found in other mosses if relative constant moisture and low pH are present [8], [12], [13]. Both the arcellinid amoebae, which are characterized by the presence of a test (syn. shell) and the Sphagnales mosses (Plantae: Sphagnopsida) have an ancient fossil record of hundreds of millions of years [14], [15]. Despite this long-lasting persistence of the Sphagnales, the radiation of the current diversity (ca. 300 species) occurred recently in the Miocene [16]. It is inferred that *Sphagnum*-dominated peatlands are as recent as the radiation of *Sphagnum* mosses.

The presence of a test in arcellinids makes them the only lineage of amoebozoans with a rich and well-preserved fossil record, which offers a rare opportunity to calibrate this part of the tree of eukaryotes. However, despite being one of the oldest lineages of eukaryotes in the fossil record (ca. 750 Myr fossils; [15]), the evolutionary history and diversification of arcellinids have not been studied in detail. It is unclear whether the high diversity of arcellinids, and in particular the Hyalospheniidae [17], reflects ancient and/or recent diversification events. Also, the taxonomic assignments and phylogenetic positions of arcellinid fossils remain largely ambiguous and are subject to much debate [1], [2], [18]. Although a putative arcellinid fossil of ca. 750 Myr has been previously used to reconstruct divergence times [1], [2], representatives of the closest sister groups to arcellinids (Amoebidae, Hartammellidae and Poseidonida) have not been incorporated into previous analyses. Therefore, the 750 Myr old fossil has not strictly been assigned to the Arcellinida but instead to nodes within the Tubulinea. Ages much younger than the 750 Myr fossils have been recently inferred for arcellinids in analyses of amoebozoan divergence times using the closest sister groups to arcellinids (e.g., *Nolandella*; [19]).

The aim of the present study is to reconstruct the evolutionary history of arcellinids, with special emphasis on the diversification of the Hyalospheniidae. After careful examination of arcellinid fossils, we reconstructed divergence times in these testate amoebae using a robust phylogeny inferred from multiple genetic markers. Moreover, we compared the diversification pattern recovered within the Hyalospheniidae, particularly in species very abundant in *Sphagnum*-dominated peatlands such as *Hyalosphenia papilio*, with the pattern of diversification in *Sphagnum* mosses. A mitochondrial cytochrome c oxidase subunit 1 (COI) dataset, including all publicly available and new sequences, was used to reconstruct the diversification pattern within the Hyalospheniidae. The 99% sequence similarity threshold and the Generalized Mixed Yule Coalescent (GMYC) model [20], [21] were used to identify evolutionarily independent units (i.e., putative species) for different morphospecies within the Hyalospheniidae.

## Materials and Methods

### 2.1 COI amplification and sequencing

Mitochondrial cytochrome c oxidase subunit I gene (COI) fragments were obtained from a total of eighteen single cells of the morphospecies *Arcella* sp (Figure S1A), *Hyalosphenia papilio*, *Nebela collaris*, *N. flabellulum*, *N. marginata* (Figure S1C–E), and one unidentified *Nebela* morphospecies (Figure S1B). The single cell specimens were isolated, amplified by semi-nested PCR, and sequenced as described in Heger and colleagues [22]. Single cell specimens were isolated either from *Sphagnum* moss or litter samples (Table S1). The specific and universal primers used in this study are listed in Table S2. COI sequences were deposited in GenBank with the accession numbers KJ544147- KJ544164. Five of the single cells that we sequenced were also characterized with light microscopy (Figure S1).

### 2.2 Dataset constructions

Reconstruction of divergence times in arcellinids was carried out using two datasets. The first matrix consisted of five concatenated proteins (i.e. actin, alpha-tubulin, beta-tubulin, eEF2 and 14-3-3) plus small subunit (SSU) rDNA sequences and corresponds with the alignment recently published by Lahr and colleagues [4]. This alignment contains a total of 53 tubulinean taxa (including 29 arcellinids). In order to distinguish the signal from these two sources, two sub-matrices were analyzed: one consisted of the five proteins for 30 taxa (including 19 arcellinids) and the other one consisted of SSU rDNA sequences for 49 taxa (including 24 arcellinids).

The second matrix consisted of COI sequences which were obtained from three different sources: (i) 16 Hyalospheniidae isolates and two new sequences from *Arcella* sp. (Table S1); (ii) 85 additional isolates within the Hyalospheniidae from Kosakyan and colleagues [23], [24]; and (iii) 298 isolates of the morphospecies *Hyalosphenia papilio* from Heger and colleagues [22]. These 401 COI sequences (Table S1 and Table S3) were manually aligned into the Kosakyan and colleagues' [24] alignment with the program Bioedit 7.0.9 [25]. When several sequences were identical or differed only by the presence of unknown nucleic acid residues (N), only the longest sequence was kept. The final alignment comprised 125 COI sequences.

### 2.3 Phylogenetic reconstructions of the COI dataset

Molecular phylogenetic analyses were performed on the 125-taxon alignment. Tree reconstructions were conducted using Maximum Likelihood (ML) analyses using Treefinder [26] and Bayesian inference methods using MrBayes v. 3.1.2 [27] as described in Heger and colleagues [22]. The Modeltest program [28] identified the general-time-reversible model with invariable sites and gamma distribution (GTR+I+G) as the most appropriate model of sequence evolution. Inferred trees were rooted with the two new *Arcella* COI sequences, an inferred sister group of the Hyalospheniidae [29]. ML analyses were run for 500 replicates. Trees were viewed using FigTree (program distributed as part of BEAST: http://tree.bio.ed.ac.uk/software/figtree/).

### 2.4 Species delimitation

**Figure 1:**
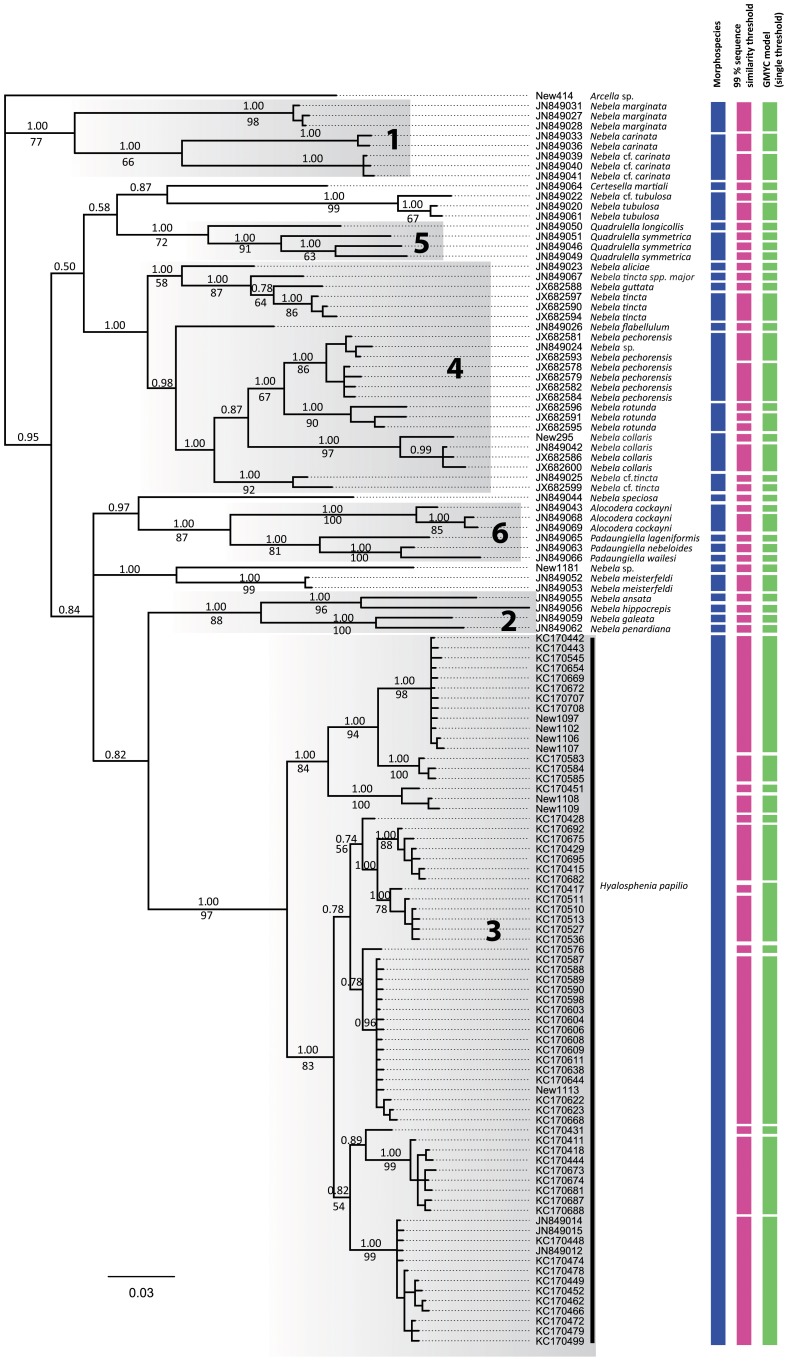
Bayesian tree of 124 COI sequences from within the Hyalospheniidae (outgroup specimens excluded). Bayesian posterior probability ≥0.5 and Bootstrap values ≥50% are given above and beneath the node, respectively. The first column on the right (green boxes) clusters individuals according to the morphology, the second column according to the 99% sequence similarity and the third according to the single threshold GMYC model. The grey boxes represent the six main clades described by Kosakyan and colleagues [24].

We identified genetic lineages with the Hyalospheniidae based on a 99% sequence similarity threshold, as suggested by Heger and colleagues [22]. Sequence divergences were calculated using the Kimura 2-parameter [30] in R, version 3.01 [31], using the package ‘ape’, version 3.0-1. Subsequently, we used the General Mixed Yule Coalescent (GMYC) model as a complementary species criterion [20], [21]. The GMYC method is based on the shift in the branching rate of the phylogenetic tree [20], [32], [33]. GMYC analyses were performed as described by Heger and colleagues [22]. The performance of the single threshold [20] and multiple threshold model [33] did not differ significantly, as revealed by the Chi-squared comparison test (chi square = 4.419, d.f. = 3, P = 0.219; Figure 1).

### 2.5 Reconstruction of divergence times and fossil assignment

Divergence times were reconstructed using BEAST v1.6 [34]. BEAST analyses on the five proteins plus SSU rDNA used the WAG+G and GTR+G models of substitution, respectively. For the COI dataset, the GTR+G model was used following the Kosakyan and colleagues [24] analyses. Uncorrelated lognormal (UCLN) model allowing different rates in different branches of the tree was applied. All analyses were run for 20×10^6^ generations, of which 5 million were discarded as burn-in. All fossils were constrained applying a uniform distribution. Different combinations of fossils were explored as described below.

**Figure 2:**
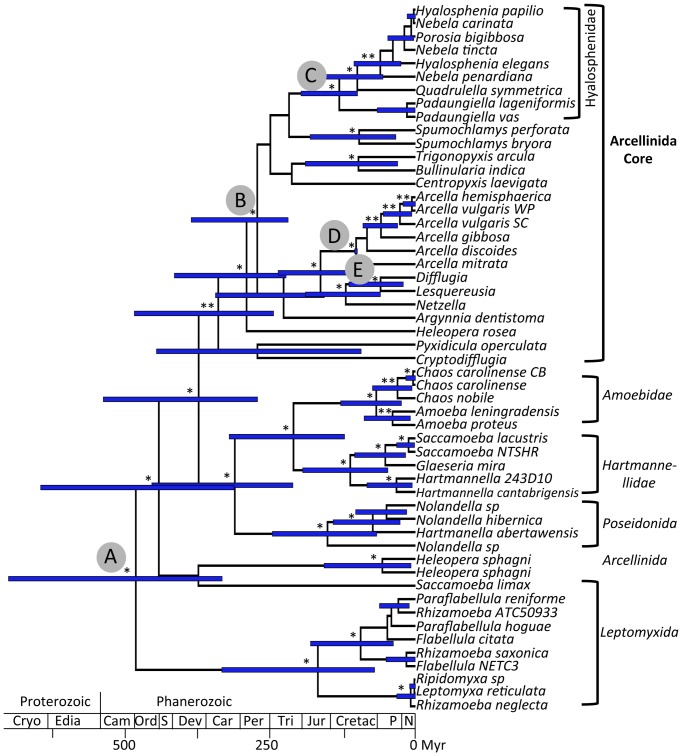
Maximum credibility tree of the Tubulinea based on 5 proteins and SSU rDNA. The chronogram is based on five proteins (Actin, alpha-tubulin, beta-tubulin, eEF2 and 14-3-3) and SSU rDNA sequences from the alignment of Lahr and colleagues [4]. Reconstructed ages and 95% Highest Posterior Density (HPD; indicated with horizontal blue bars) were retrieved using BEAST [34]. Letters on nodes correspond to calibration points used, i.e. A = Arcellinids, B = *Centropyxis*, C = Hyalospheniidae, D = *Arcella* (see Table 2). Calibration E* = Difflugia* and *Lesquereusia* is used in alternative analyses. Taxonomic assignment is indicated on the right. Geological timescale is indicated at the bottom: Cryogenian, Ediacara, Cambrian, Ordovician, Silurian, Devonian, Carboniferous, Permian, Triassic, Jurassic, Cretaceous, Paleogene and Neogene. One asterisk correspond to posterior probabilities (PP) = 1 and two asterisk is for PP>0.9.

Fossil calibration was applied to five nodes where 10 fossil records could be assigned. These 10 fossil records correspond to two arcellinids, two *Arcella*, two *Centropyxis*, one hyalospheniid, two *Difflugia* and one *Lesquereusia*; all were selected from a wide variety of publications (Table 1, Table S4). An age of 770 Myr from the oldest arcellinid fossils (770-742 Myr; [15]) was used on the root as a maximum bound (node A; Figure 2), which allows considering the second oldest record (662 Myr, [35]). This node includes non-arcellinid taxa but phylogenetic analyses have revealed that arcellinid testate amoebae are paraphyletic within the Tubulinea [4]; therefore, we used it only as a maximum age constraint. A conservative age for the *Arcella* fossils was used for its crown node (node D; Figure 2) at a minimum bound of 100 Myr (mid Cretaceous; [36]) and a maximum bound of 5% older (105 Myr). Because the phylogenetic position of *Centropyxis* is not resolved [4], its fossil age (220 Myr, Carnian within the Triassic, [37]) was assigned to the first well-resolved node containing *Centropyxis* as a minimum age (node B; Figure 2) and a maximum bound equal to the root age (770 Myr). The Hyalospheniidae fossil (node C; Figure 2) was used as a conservative minimum bound (100 Myr, Albian–Cenomanian, within the Cretaceous; [38]) and a maximum bound equal to the root age (770 Myr) was applied in order to explore the uncertainty of the age of this clade. Two taxa represented in our dataset, *Difflugia* and *Lesquereusia*, could be used for the assignment of two fossils from the lower Albian (Cretaceous; [39], [40]). *Difflugia* is a paraphyletic group [41], which leads to uncertainty. With this in mind, we explored the effect on the initial calibration scheme of constraining the split of *Difflugia-Lesquereusia* (node E) to a conservative minimum age of 100 Myr and a maximum age equal to the root (770 Myr). In addition, to evaluate the accuracy of nodes D and E, the *Difflugia-Lesquereusia* calibration (node E >100<105 Myr) was combined with a relaxation of *Arcella* upper bound (node D) to an age equal to the root. All these calibrations were combined into three different analyses to check congruency among them (Table 2).

**Table 1:** Reference of relevant fossil records and calibrations used in this study.

Taxa	Node	Period	Calibration (Myr)	Reference
Arcellinida	A	Neoproterozoic, Cryogenian	ca. 662	[35]
Arcellinida	A	Neoproterozoic	<770-742	[15]
*Centropyxis*	B	Carnian (Triassic)	>220	[37]
Hyalospheniidae	C	Albian– Cenomanian (Cretaceous)	>100	[38]
*Arcella*	D	Mid Cretaceous	>100	[36]
*Difflugia*	E	Lower Albian (Cretaceous)	>100	[40]
*Lesquereusia, Difflugia*	E	Late Albian (Cretaceous)	>100	[39]
*Arcella, Centropyxis Difflugia, Lesquereusia*	B, D, E	Early Permian	>299	[49]
*Arcella, Centropyxis Difflugia*	B, D, E	Late Carboniferous–Early Permian	>299	[42]

Taxonomic assignment, geological time and period are taken from the references (right column). Placement of the fossil in each corresponding node (see Figure 2) is indicated. Time of the fossil record is in Myr (million years).

**Table 2:** Divergence times from 5 proteins +SSU rDNA dataset under different calibration schemes.

	Root	Arcellinid core	Hyalospheniidae	*Arcella*-Hyalospheniidae
Four fossils (A, B, C, D)	483	341	132	273
	(334–703)	(245–486)	(100–198)	(220–386)
Five fossil (A, B, C, D), *Difflugia* (E) >100-<770	534	378	141	305
	(381–749)	(270–527)	(100–216)	(220–424)
Five fossil (A, B, C), *Difflugia* (E) >100<105 and *Arcella* (D) >100<770	568	400	145	326
	(408–766)	(286–571)	(100–228)	(229–459)
Four fossils (A, C, D) *Centropyxis* (B) >299 <770	573	409	146	332
	(440–757)	(324–529)	(100–225)	(299–417)

Divergence times for major clades are indicated in Myr (million years). Capital letters represent fossil calibrations (upper and lower bounds) for the corresponding node (see Figure 2): A) root <770 Myr, B) *Centropyxis* clade >220<770 Myr, C) Hyalospheniidae >100 <770 Myr, D) *Arcella* >100 <105 Myr, unless otherwise specified and E) corresponds to the node *Difflugia-Lesquereusia.*

In order to evaluate three recently published fossils records, we also explored alternative fossil age assignments of 299 Myr for (i) *Arcella* (node D), (ii) *Centropyxis* (node B) and (iii) *Difflugia* and *Lesquereusia* (node E); we examined each of them independently following two recent reports [42]. Because this new calibration is three times older than the initial calibrations for *Arcella* and *Difflugia*, an upper bound of 5% older (314 Myr) was applied in both analyses. In light of the fact that preliminary analyses recovered the maximum bound for the root (ca. 770 Myr), we allowed a relaxation of the root age to <3000 Myr.

**Figure 3:**
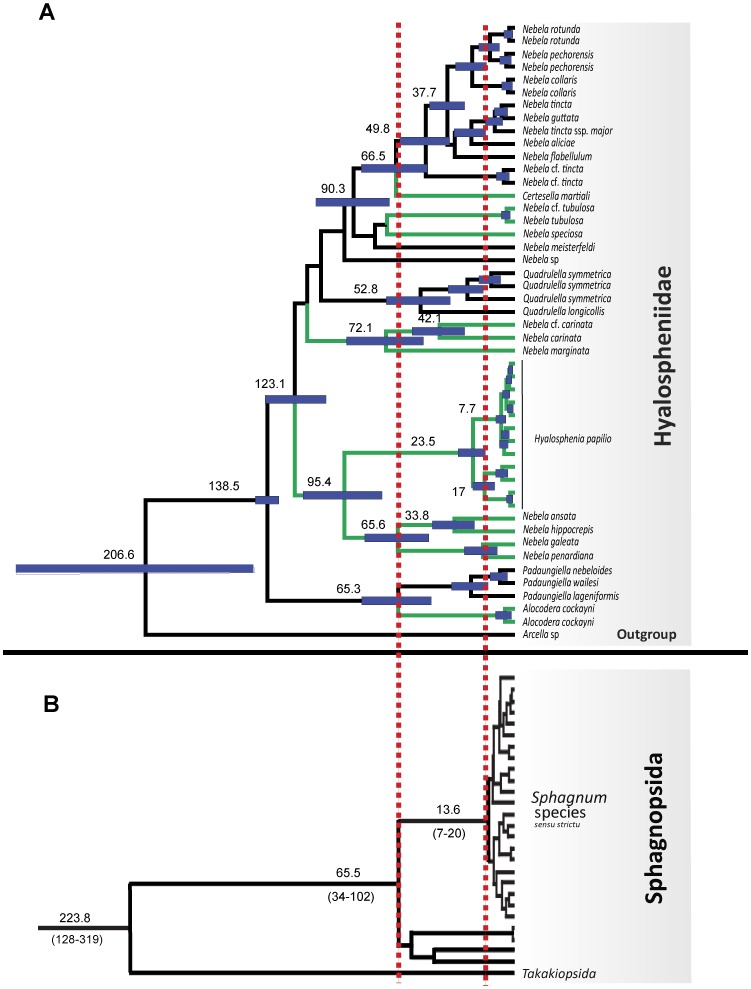
Maximum credibility tree of Hyalospheniidae obtained from COI dataset analyses. A. Reconstructed ages and 95% Highest Posterior Density (HPD; indicated with horizontal blue bars) were retrieved using BEAST [34] with only one COI sequences per GMYC lineage (i.e. evolutionarily independent unit). Arcellinid lineages occurring only in *Sphagnum* dominated peatlands are marked with green lines. Lineages that occur in *Sphagnum* dominated peatlands and/or other habitats are marked with black lines. Numbers on nodes correspond to divergence times in million years. Taxonomic assignment is indicated on the right. B. *Sphagnum* chronogram from eight mitochondrial, plastid, and nuclear genes. Dates (±standard errors) from Shaw and colleagues [16]. Red dotted vertical lines indicate early split of the Sphagnopsida (65.5 Myr ago) and radiation of ca. 300 spp of *Sphagnum* (13.6 Myr ago).

For the COI marker analysis, the alignment comprises one sequence from each evolutionarily independent lineage detected with the GMYC model. The Hyalospheniidae crown age was calibrated using an age range reconstructed from the result of the three main analyses of arcellinids (Table 2; Figure 3). These results were used as minimum and maximum bounds applying a uniform distribution. The root between *Arcella* and the Hyalospheniidae was constrained to a reasonable maximum bound of 500 Myr (five times older than its fossil record). Inclusion of *Arcella* as the outgroup in the COI analysis allowed us to evaluate the *Arcella* - *Hyalospheniidae* split with results from the concatenated dataset.

### 2.6 Diversification rates and saturation analyses

A model fit to speciation with one, two or three diversification rates was tested with LASER [43]. This program compares birth-death models with various rates of diversification against the null expectation of a constant rate. LASER was used to identify when a given shift on diversification rate occurred in our GMYC species chronograms. One hundred trees were randomly taken from the GMYC Highest Posterior Density trees (HPD) and analysed with LASER. The critical Delta AICrc value was estimated from simulations under rate-constant model. Lineage-Through-Time (LTT) plots were drawn for these chronograms. The overall diversification rate assuming an extinction between 0 and 0.9 was estimated with Magallon and Sanderson [44] equation 7 as implemented in R version 3.01 [31]. The program DAMBE [45] was used to analyze nucleotide saturation in our dataset. No saturation was observed for the COI data set (data not shown).

## Results

### 3.1 New sequences obtained in this study

Eighteen new COI sequences were obtained for the present study (i.e., two *Arcella* sp. and 21 isolates within the Hyalospheniidae; Table S1). The obtained COI fragment lengths ranged from 338 bp to 631 bp. The two *Arcella* sp. sequences represented the first arcellinid COI sequences that did not belong to the *Hyalospheniidae.* Both *Arcella* sp. sequences were identical even though they were isolated from different regions.

### 3.2 COI phylogeny and species delimitation

Bayesian and Maximum Likelihood analyses were congruent with each other, with only a few exceptions. Our phylogenetic tree revealed six main clades (Figure 1), as reported in Kosakyan and colleagues [24]. Bayesian analyses provided the highest posterior probabilities (PP) for these six lineages (i.e., 1.00 PP), and Maximum Likelihood analyses provided strong to moderate statistical support, except for clade 4, which is not supported (Figure 1). Besides these six clades, a few species had an uncertain affiliation, such as the newly sequenced *Nebela* sp. (Figure S1B). Based on comparative morphology, a total of 26 morphospecies were identified. Sequences from the same morphospecies formed a monophyletic group, except the sequences from the *N. tincta* complex (Figure 1). The number of non-identical sequences per morphospecies varied from one to seventy one (i.e., *Hyalosphenia papilio*). Based on a 99% sequence similarity threshold, forty-nine lineages were identified. Similarly, the General Mixed Yule-coalescent (GMYC), based either on the single or multiple threshold model, revealed a total of forty-seven entities. Within the *H. papilio* morphospecies, which has been intensively sampled, both the 99% sequence similarity threshold and the GMYC model revealed an important number of putative species (13 and 12, respectively). Overall, the 99% sequence similarity threshold and the GMYC model provide convincing support for the identification of Evolutionarily Independent Units within the Hylalospheniidae [22], [46] (Figure 3).

### 3.3 Fossil calibrations in arcellinid testate amoebae

The validity of the fossil records was evaluated by reconstructing divergence times with different combinations of fossils. The reconstructed age of the Hyalospheniidae is robust across different calibration schemes (Table 2). The original scheme of four fossils gives an age of 132 (100–198) Myr for the Hyalospheniidae. For *Arcella*, *Centropyxis*, and *Difflugia*-*Lesqueresia*, two alternative fossil ages were used. The use of two different combinations of the upper bounds of the fifth fossil *Difflugia*-*Lesqueresia* (node E; 100 Myr) with *Arcella* fossil (node D; 100 Myr) retrieved similar ages overall to the four fossils scheme (Table 2): 141 (100–216) and 145 (100–228) Myr for the Hyalospheniidae. The original scheme using 220 Myr for *Centropyxis* also recovered similar ages to the alternative scheme using 299 Myr (Table 2): 132 (100–198) vs 146 (100–225) Myr for the Hyalospheniidae, respectively; 483 (334–703) vs. 573 (440–757) Myr for the root, respectively. In contrast, the use of an alternative age of 299 Myr vs 100 Myr for *Arcella* and for *Difflugia*-*Lesquereusia* gave unrealistic ages given the oldest undisputed fossil records of eukaryotes: root 1098 (685–1550) Myr and 1213 (814–1700) Myr (Table 2 and Table S5). Therefore, the 299 Myr fossil record of *Arcella* and *Difflugia*-*Lesquereusia* was not taken into account. Overall, the original scheme of four fossils gives slightly narrower confidence intervals and the incorporation of the 299 Myr fossils for *Centropyxis*, *Arcella* and *Difflugia*-*Lesquereusia* clade retrieved older ages.

### 3.4 Reconstruction of divergence times in Arcellinida

Our reconstruction of divergence times using five calibration points recovered an age of arcellinids *sensu stricto* (i.e. core Arcellinida) of 341–409 (245–529) Myr (Table 2). The age of the root, in this study considered as Arcellinida *sensu lato* was reconstructed at 483–573 (334–757) Myr. An age similar to the oldest fossils attributed to arcellinids of 770–742 Myr [15] or alternatively 662 Myr [35] was only reached when using unrealistic fossil record ages like 299 Myr for *Arcella*, which recovered an age of over 1000 Myr for the root (Table S5). By contrast, the age reconstructed for the Hyalospheniidae of 132–146 (100–225) Myr from multiple fossil combinations predates its oldest fossil record (ca. 100 Myr; Albian–Cenomanian, [38]) that was used as minimum bound in the reconstructions.

Results from the concatenated dataset were in agreement with both the five-protein dataset alone and SSU rDNA dataset alone. The root of the tree was 483 (334–703) Myr for the concatenated dataset based on the original calibration scheme (Table 2 and Table S5), 475 (289–705) Myr for the five-protein dataset and 429 (299–643) Myr for the SSU rDNA dataset. When considering the confidence intervals, the SSU rDNA sequences retrieved slightly younger ages (Table S5). The main difference between the concatenated and the two partitioned analyses was the absence of *Centropyxis* in the five-protein dataset; therefore, the confidence intervals for its corresponding node (node B) recovered a lower bound of 147 Myr (while fossil dates are 220 Myr). The main incongruence between the two partitioned analyses and the concatenated analyses comes from the Hyalospheniidae clade that is reconstructed at 170 Myr in the analysis of the five-protein dataset (Table S5). This was even more striking because the basal split within the Hyalospheniidae (between *Padaungiella* and the rest of Hyalospheniidae) was not present in the five-protein dataset. The age of this node (170 Myr) correponded to 89 Myr in the analysis of SSU rDNA and 100 Myr in the analysis of the concatenated dataset. This discrepancy reflected the high proportion of missing data and resulting long branches found for *Nebela carinata* (170 Myr; Table S5) in the five-protein analysis as compared to the concatenated analysis (Figure 2); the three remaining species of Hyalospheniidae split 60 Myr ago.

### 3.5 Diversification of Hyalospheniidae from COI marker

**Figure 4:**
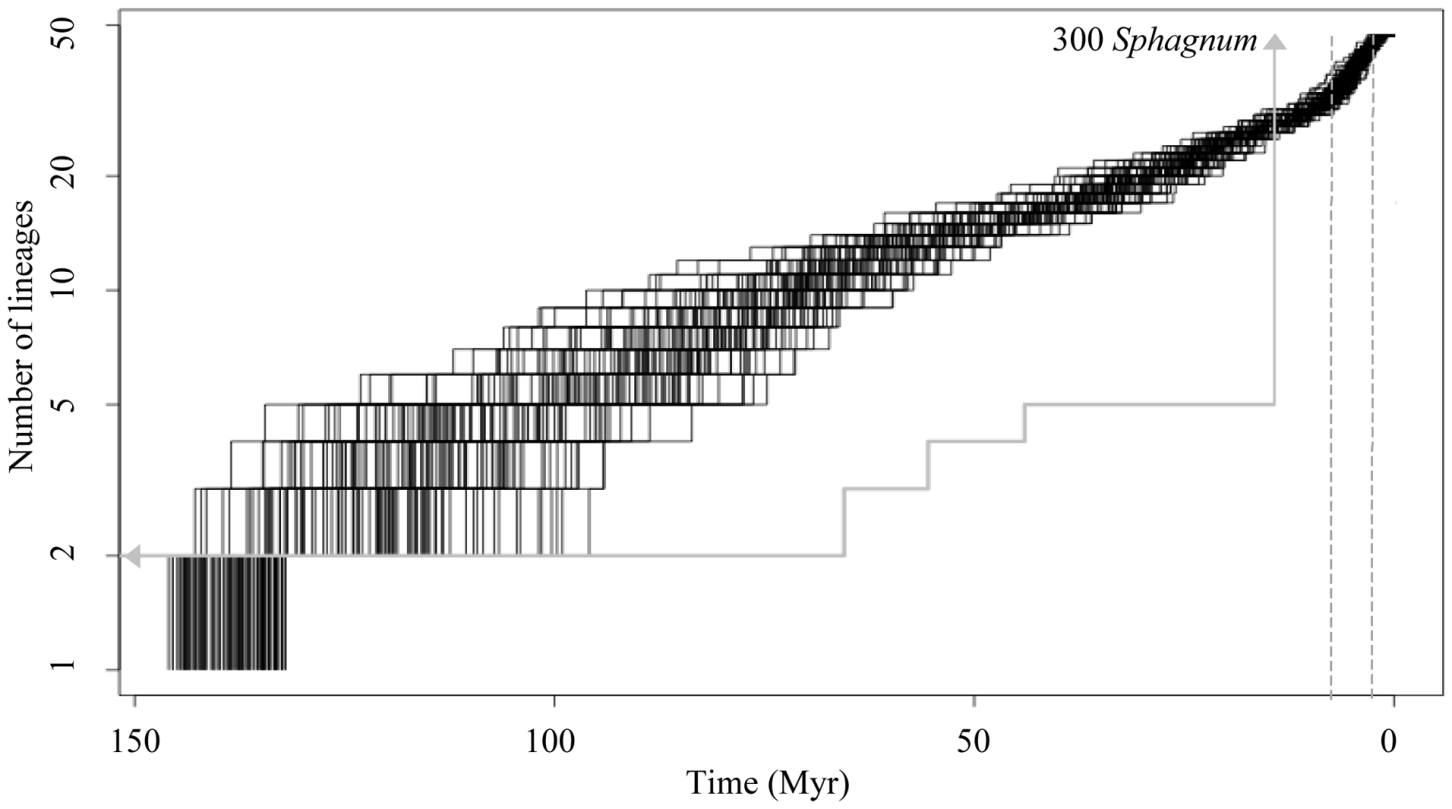
Lineage-Through-Time (LTT) plot of Hyalospheniidae. Lineage-Through-Time (LTT) was plotted using 100 trees of the Hyalospheniidae from the COI dataset analyses. The number of lineages (x axis) is plotted against time (y axis) in such way that each increase on the number of lineages represents a cladogenesis (a node) in one of the 100 phylogetic trees. These 100 trees were randomly taken from Highest Posterior Density (HPD) generated in BEAST from the GMYC dataset. Diversification rate shifts leading to a rate increase (7 Myr ago) and a rate decrease (2.5 Myr ago) as detected in LASER are indicated with dotted grey lines. Grey LTT line is for mosses as extracted from the tree in Shaw and colleagues [16]. The radiation of 300 *Sphagnum* at 14 Myr ago is indicated with a vertical line and an arrow. Grey arrow on the left indicates the stem age of *Sphagnum* at 223.8, Shaw and colleagues [16].

A constant diversification was observed in the Lineage-Through-Time (LTT) plots of the Hyalospheniidae from the crown age until ca. 10 Myr from (Figure 4). Analyses of 100 trees randomly taken from the GMYC Highest Posterior Density trees (HPD) recovered a mean shift time leading to a rate increase 7 Myr ago (95% CI = 4.9–10.3) followed by a rate decrease 2.5 Myr ago (95% CI = 2.1–10.3). Forty-five out of 100 trees showed a Delta AICrc higher than the critical Delta AICrc value (4.15) for the yule-3-rates against the best rate constant birth-death model. The overall diversification rate from the GMYC trees varied between 0.023–0.012 (extinction 0 and 0.9, respectively).

The analysis using the GMYC model demonstrated a high proportion of evolutionarily independent units older than 7 Myr. However, evenly distributed cladogenesis in the last 25 Myr was only seen for three morphospecies complexes: *Quadrulella symmetrica*, *Nebella tincta sensu lato* and *Hyalosphenia papilio*. The new samples sequenced in this study recovered two new cladogenetic events of <7 Myr for *N. tincta* (New 295 = KJ544161) and *H. papilio* (New 1108 = KJ544152 and New 1109 = KJ544153) and a new cladogenesis event of >90 Myr for *Nebela* sp. (New 1181 = KJ544164). However, inclusion of more samples not always means ancient cladogenesis (e.g., nine sequences of *N. marginata* recovered only one cladogenesis event of <1 Myr; results not shown).

## Discussion

### 4.1 A temporal scale for arcellinid diversification

Despite being the lineage of amoebozoans with the richest fossil record, the assignment of the oldest arcellinid fossils to a phylogenetic node is controversial [1], [2], [19]. In this study, we found that the oldest amoebozoan fossil (770-742 Myr; [15]) cannot be assigned with certainty to the Arcellinida *sensu stricto* (i.e., the core Arcellinida). However, the paraphyly of arcellinids allows us to consider an assignment of the oldest fossil to deeper nodes. Therefore, when we took into account the paraphyly of the arcellinid testate amoebae, as recently suggested by Lahr et al. (2013), we were able to reconcile conclusions based on the fossil record and molecular clock analyses.

Two studies have recently discussed reconstructed ages of arcellinids (attributed to 770-742 Myr; [15]) while referring to a different node. In the case of Parfrey et al. (2011), the node linked arcellinids and *Rhizamoeba*, (not the most closely related lineages, which are Amoebidae, Hartammellidae and Poseidonida); in the case of Berney and Pawlowski (2006), the node was within the Tubulinea (i.e., the node linking *Hartmanella* and *Echinamoeba*). The age reconstructed by Berney and Pawlowski [1] (ca. 600 Myr) is consistent with our results for the Tubulinea [483–573 (334–757) Myr]. In Parfrey et al. [2], the age reconstructed for the divergence between arcellinids and *Rhizamoeba* is ca. 1000 Myr when using Proterozoic fossils. However, Parfrey et al. [2] inferred ages of ca. 600 Myr when they only used more reliable (undisputed) Phanerozoic fossils. Our results do not conflict with these two previous studies nor with the fossil record inferred by Porter and Knoll ([15]; 770-742 Myr) and Bosak and colleagues ([35]; 662 Myr). When considering all of these studies together, we think that the most conservative interpretation of the oldest amoebozoan fossils is to assign them to the Tubulinea, which comprises many naked amoebae. This interpretation is further supported by a recent study on divergence times for Amoebozoa where arcellinids and the closest sister groups have been incorporated and that recovered ca. 600 Myr for Tubulinea [19]. This leads us to infer that either arcellinid testate amoebae appeared several times independently during the evolution of the Tubulinea or the test was independently lost in different lineages from a common ancestor with a test. The first scenario would be favored when considering the eukaryophagy hypothesis [47], [48].The high number of eukaryophagic protist fossils in the mid-Neooproterozoic may have triggered the radiation of tests in amoebozoans and other protists [47] because tests provide physical protection against predators.

The other amoebozoan fossils (Table 1 and Table S4) were in agreement with the molecular clock reconstructions. After careful examination of 24 studies, we selected 10 fossils (including the two oldest arcellinid fossils) and assigned them to five nodes for reconstruction of divergence times. The other 14 fossils (Table S4) reported in these studies were not incorporated into our analyses because either they have been previously dismissed (five fossils), the taxonomic assignment was uncertain (two fossils), or they did not correspond to the oldest fossil of the group (Table S4). In our analyses, the most incongruent results were obtained when incorporating *Arcella*, *Difflugia* and *Lesqueresia* fossils of 299 Myr [42], [49]. If these lineages are indeed that old, then our molecular clock reconstructions using these fossils (>1100 Myr for split of *Rhizamoeba* from arcellidins; Table S5) would be incongruent with both previous molecular studies and with undisputed fossil records assigned to deep nodes of eukaryotes [1], [2], [19]. If the rate of molecular evolution decelerated in *Arcella*, *Difflugia* and *Lesqueresia*, then using external calibrations would result in younger divergence times than expected (299 Myr). By contrast, if we calibrated these nodes with ancient dates (299 Myr fossils) and assumed a rate deceleration, then all of the inferred ages would be pushed to the past (Table S5).

### 4.2 Diversification of two ancient lineages: The Hyalospheniidae (Amoebozoa: Arcellinida) and *Sphagnum* mosses (Plantae: Sphagnopsida)

Based on our reconstructed divergence time analyses, the diversification of arcellinids during the Phanerozoic is the most likely scenario (Figure 2). The age recovered for arcellinids *sensu stricto* (core Arcellinida: 341–409 (245–529) Myr; Table 2) is in agreement with divergence time analyses that used fossils from outside the Tubulinea (arcellinid stem age: 254–522 Myr; [19]). This is the time when land plants colonized terrestrial soils with the oldest records of spores of ca. 470 Myr [50]. Among the oldest lineages of land plants are *Sphagnum* with a fossil record of 330 Myr [14] and with slightly older divergence times from molecular phylogenetic analyses (380 Myr; [51]). Because several taxa within the Hyalospheniidae exclusively, or almost exclusively, inhabit *Sphagnum*-dominated peatlands (Figure 3), one can expect that the *Sphagnum* lineage and the Hyalospheniidae lineage (or sublineages) have experienced similar diversification dynamics through time, especially if a longstanding ecological interaction between both groups of organisms has occurred. Hubers and Kerp [14] pointed out a similar ecological role between Carboniferous (359-299 Myr ago) protosphagnales and modern *Sphagnum* in peatbogs. However while *Sphagnum* shows a diversification stasis from ca. 220 to ca. 14 Myr (with only one cladogenesis; [16]; Figure 3), the Hyalospheniidae seem to follow a more constant diversification pattern from ca 140 Myr until very recent times (ca. 7 Myr) when they increased the rate of diversification (Figure 3 and Figure 4).

The Cretaceous (145-65 Myr ago) was the beginning of a great change in terrestrial ecosystems [52]. The age reconstructed for the diversification of Hyalospheniidae matches precisely with the origin and diversification of angiosperms (ca 130 Myr ago, [53]) as well as the diversification of most mosses [52]. Therefore, the Hyalospheniidae certainly not only depend on the diversity of *Sphagnum* mosses but also on the diversification of different terrestrial habitats with different lineages of modern mosses and angiosperms. The relationship between *Sphagnum* and some of the Hyalospheniidae clades occurring only in peatlands should be interpreted as a recent specialization to the *Sphagnum-*dominated peatland ecosystem during the Miocene (5–20 Myr ago; [16]).

The radiation of the Hyalospheniidae detected here (7 Myr ago) occurs only after the diversification of *Sphagnum* (14 Myr ago; [16]). However, the diversification pattern found in some parts of the tree suggests that a deeper taxonomic sampling is required to improve our understanding of these ecological relationships. Long branches can indicate either long periods without diversification (because of high extinction or low speciation rates) or poor sampling. The inclusion of additional sequences from *H. papilio* (a single morphospecies) in our molecular phylogenetic analyses demonstrated its diversification in the last ca. 20 Myr. Expanding the sequence sampling (even if taxonomic diversity is still awaiting description) may lead to a pattern of similar diversification in other parts of the Hyalospheniidae tree. This would produce a better fit between the diversification of the Hyalospheniidae amoebae and *Sphagnum* during the Miocene. If other *Sphagnum*-associated morphospecies within the Hyalospheniidae, for instance, had a diversification pattern similar to the one described for *H. papilio*, then the hypothesis that the establishment of *Sphagnum* dominated-peatland ecosystem influenced the diversification of clades within the Hyalospheniidae would be supported. Although currently unclear, it is also possible that the presence of arcellinid testate amoebae may have in turn influenced the diversification of *Sphagnum* mosses.

## Supporting Information

Figure S1 **Light micrographs illustrating six arcellinid specimens we isolated for single-cell PCR.** A: *Arcella* sp (Genbank KJ544162), B: *Nebela* sp. (Genbank KJ544164), C–E: *Nebela marginata* (Genbank KJ544160, KJ544156 and KJ544157). A and C–E specimens were sampled from *Sphagnum*-dominated peatland while B was sampled from forest litter. Scale bars = 50 µm.
(TIF)

Table S1 **List of the Hyalospheniidae single cells from which COI sequences were obtained.**
(XLS)

Table S2 **Sequences of the primers used in this study.**
(XLS)

Table S3 **List of publicly available COI sequences from the Hyalospheniidae.**
(XLS)

Table S4 **References of the fossil records not considered in this study.**
(XLS)

Table S5 **Divergence times from 5 protein + SSU datasets combined and from 5protein and SSU independently under different calibration schemes.** Divergence times for major clades are indicated in Myr (million years). Capital letters represent fossil calibrations (upper and lower bounds) for the corresponding node (Figure 2): A) root <770 Myr, B) *Centropyxis* >220<770 Myr, C) Hyalospheniidae >100<770 Myr, D) *Arcella* >100<105 Myr, unless otherwise specified; * In the 5 proteins dataset, Hyalospheniidae did not include the basal split between *Padaungiella* and the rest of Hyalospheniidae. E) corresponds to the node *Difflugia-Lesquereusia*.
(XLS)
