# Supplementary figures and images for: Old Lineages in a New Ecosystem: Diversification of Arcellinid Amoebae (Amoebozoa) and Peatland Mosses
Source: PLoS One. 2014 Apr 24;9(4):e95238. doi: 10.1371/journal.pone.0095238 (PMC3999201; doi:10.1371/journal.pone.0095238)

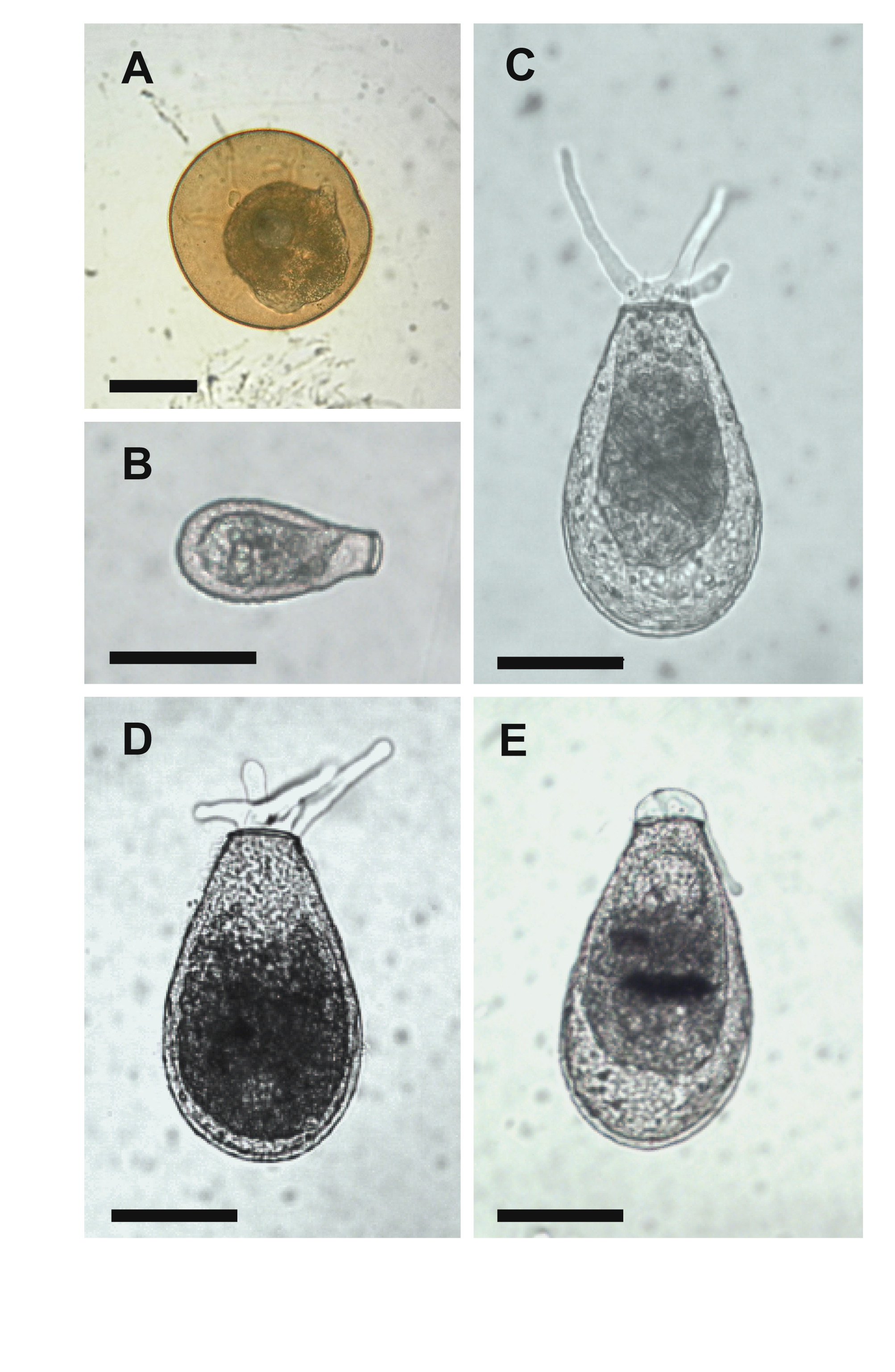

Supplement: Figure S1 — Light micrographs illustrating six arcellinid specimens we isolated for single-cell PCR. A: Arcella sp (Genbank KJ544162), B: Nebela sp. (Genbank KJ544164), C–E: Nebela marginata (Genbank KJ544160, KJ544156 and KJ544157). A and C–E specimens were sampled from Sphagnum-dominated peatland while B was sampled from forest litter. Scale bars = 50 µm. (TIF) [file pone.0095238.s001.tif]
